# Impact of early changes in serum biomarkers following androgen deprivation therapy on clinical outcomes in metastatic hormone-sensitive prostate cancer

**DOI:** 10.1186/s12894-018-0353-4

**Published:** 2018-05-08

**Authors:** Hiromi Sato, Shintaro Narita, Norihiko Tsuchiya, Atsushi Koizumi, Taketoshi Nara, Sohei Kanda, Kazuyuki Numakura, Hiroshi Tsuruta, Atsushi Maeno, Mitsuru Saito, Takamitsu Inoue, Shigeru Satoh, Kyoko Nomura, Tomonori Habuchi

**Affiliations:** 1 Department of Urology, Akita University School of Medicine, 1-1-1 Hondo, Akita, 010-8543 Japan; 2 Department of Urology, Yamagata University School of Medicine, Yamagata, Japan; 3 Center for Kidney Disease and Transplantation, Akita University Hospital, Akita, Japan; 4 Department of Public Health, Akita University School of Medicine, Akita, Japan

## Abstract

**Background:**

Less evidence is known about the role of early changes in serum biomarker after androgen deprivation therapy (ADT) in patients with metastatic hormone-sensitive prostate cancer (mHSPC). Here we evaluated the impact of pre-treatment prognostic factors and early changes in serum biomarkers on prostate specific antigen (PSA) progression-free and overall survival rates in mHSPC.

**Methods:**

We retrospectively reviewed the medical records of 60 mHSPC patients (median age 72 years) treated with ADT whose laboratory data at baseline and following 12 weeks were available.

**Results:**

Forty-four patients (73%) had PSA progression and 27 patients (45.0%) died during a median follow-up of 34 months. The multivariable Cox hazard model demonstrated that a log-transformed baseline PSA level (*p* = 0.003) and an extent of bone disease (EOD) score of ≥3 (*p* = 0.004) were statistically associated with an increased risk for PSA progression whereas one unit increase in a log-transformed PSA change (baseline-12 weeks) was associated with a decreased risk for PSA progression (*p* = 0.004). For overall survival, a high level of alkaline phosphatase (ALP) at 12 weeks was associated with increased risk (*p* = 0.030) whereas a one-unit increase in the log-transformed PSA change was associated with decreased risk (*p* = 0.001).

**Conclusions:**

An increased level of PSA at baseline, or an EOD score of ≥3 may be a good predictor of PSA progression, and a high level of ALP at 12 weeks may be a risk predictor of death. A larger decline in PSA at 12 weeks from the baseline was associated with both PSA progression-free and overall survival time. Early changes in serum biomarkers may be useful in predicting poor outcomes in patients with mHSPC who are initially treated with ADT.

## Background

Prostate cancer is the second leading cause of cancer death among men worldwide [1]. Although androgen deprivation therapy (ADT) has been a standard treatment for metastatic prostate cancer, its response and duration vary. Recent studies have shown that an early chemohormonal therapy with docetaxel results in improved overall survival (OS) than ADT alone in patients with metastatic hormone-sensitive prostate cancer (mHSPC) [2, 3]. Sweeney et al. reported that the benefit of early chemotherapy for mHSPC was more apparent in the subgroup with a high-volume disease [2]. In addition, some prostate cancer patients who received chemotherapy experienced severe toxicities, particularly in Asian patients [4, 5]. Therefore, it is important to predict a poor response to ADT in order to screen appropriate candidates for the administration of early chemotherapy combined with ADT.

Several candidate biomarkers have been found to have prognostic value in predicting poor outcomes in patients with mHSPC treated with ADT [6], and risk stratification models in mHSPC using these risk factors have been proposed [6, 7]. Furthermore, recent studies showed that dynamic changes in serum biomarkers at the cancer’s early stage, including prostate specific antigen (PSA) or alkaline phosphatase (ALP) changes, had a higher impact on clinical outcome in patients with mHSPC and/or castration-resistant prostate cancer than that of pretreatment variables [8, 9]. However, there is less evidence about the impact of early changes in serum biomarker after ADT in patients with mHSPC, and the comparison to pretreatment prognostic factors, including the categorized definition of high-tumor burden in recent trials, still needs to be investigated.

In order to predict PSA progression and poor survival in patients with mHSPC, initially treated with ADT, we assess the impact of the early changes in serum biomarkers at 12 weeks along with pretreatment prognostic factors such as a high-volume disease in the CHAARTED trial on PSA progression-free survival and OS.

## Methods

### Study population

We retrospectively reviewed the medical records of 80 consecutive primary metastatic prostate cancer patients treated with ADT at the Akita University Hospital in Japan between 2000 and 2015. Sixty patients whose laboratory data at 12 weeks post ADT were available and included in this study. Our institutional review board approved this study.

### Treatment and evaluation

All patients were treated with a maximal androgen blockage (MAB) as an initial therapy. MAB therapy consisted of a chemical or surgical castration in combination with bicalutamide. Sequential treatments after first-line ADT were administered at physician’s discretion. PSA examination was routinely performed at least every three months. Radiographic examination was routinely performed before ADT, and thereafter at physician’s discretion. PSA progression was defined according the Prostate Cancer Clinical Trials Working Group 2 (PCWG2) criteria [10]. OS was calculated as the time from ADT initiation to the date of death.

### Statistical analyses

All pretreatment variables were evaluated when prostate cancer was diagnosed. The extent of bone disease (EOD) scores for each patient was classified by bone scintigraphy according to the definition reported by Soloway et al. at initial diagnosis [11]. Eastern cooperative oncology group performance-status score (ECOG-PS) and the presence of bone pain were evaluated by inquiry and physical examination. A high-volume disease in the CHAARTED trial was defined as the presence of visceral metastases or ≥ 4 bone metastatic lesions with ≥1 beyond the vertebral bodies and pelvis, and a low-volume disease was defined as other than the definition of a high-volume disease [2].

There were missing values for the levels of Hb, ALP, and LDH at baseline (*n* = 4) and following 12 weeks (*n* = 5), and for the EOD scores (*n* = 5), and CHAARTED criteria (*n* = 5). We replaced the missing data with the mean or the median according to the distribution of the corresponding variable. Missing values for the EOD scores and CHAATED criteria were not imputed for ethical reasons. Because similar results were obtained before and after imputation, we present only the results with imputation.

PSA progression-free survival and OS curves were estimated using the Kaplan-Meier method, and the comparison of survival curves was statistically tested using a log-rank test. The subjects were split into three groups according to their disease status and PSA change and compared. The three groups were a group with a high-volume disease in the CHAARTED trial, a group with a low-volume disease in the CHAARTED trial and larger PSA change larger than or equal to the median, and a group with a low-volume disease in the CHAARTED trial and PSA change smaller than the median.

To identify independent prognostic factors for PSA progression-free survival and death, Cox proportional hazard model was applied to calculate the hazard ratio (HR) along with the 95% confidence interval (95%CI) adjusting for the following variables: patient age; biopsy Gleason score; EOD score; ECOG-PS; the presence of bone pain; the presence of visceral metastases; high- or low-volume disease in the CHAARTED trial; log-transformed baseline PSA level and log-transformed PSA change (baseline-12 weeks after ADT); low (≤ mean g/dl) or high (>mean, g/dl) Hemoglobin (Hb) level (baseline and 12 weeks after ADT); low (≤median, IU/L) or high (>median, < IU/L) ALP levels (baseline and 12 weeks after ADT); and low (≤median, IU/L) or high (>median, IU/L) LDH levels (baseline and 12 weeks after ADT). The selected cut-off points were 1 for ECOG-PS, 8 for the biopsy Gleason score, and 3 for the EOD score. The final multivariable Cox proportional hazards models were determined using PROC PHREG in statistical analysis system (SAS) and SCORE option and were based on a subset of model selection methods (i.e., stepwise, forward, and backward) using a *p* < 0.05 as entry criterion, and a *p* ≥ 0.05 as removal criterion. The best model (Model I) was determined with the highest chi-square statistic and lowest score for Akaike information criterion (AIC). Because of high correlation between a log-transformed baseline PSA level and a log-transformed PSA change (Pearson coefficient 0.979, *p* < 0.0001), Model II was built where a log-transformed baseline PSA level was removed from Model I.

All statistical analyses were performed using SPSS software version 19.0 and SAS (version 9.4, NC, USA). All reported *p*-values were two-sided, and considered statistically significant at *p* < 0.05.

## Results

The baseline patient characteristics in this study are shown in Table 1. The median age was 72 years (range, 56–93 year). Forty-four patients (73%) had PSA progression and 27 patients (45.0%) died of cancer progression (except one patient who died of other disease) during a median follow-up of 34 months. The median PSA level at pre-treatment and 12 weeks after the initiation of MAB therapy were 195 ng/ml and 2.6 ng/ml, respectively. The median percentage change in the PSA level at 12 weeks was 98.5% {Interquartile range (IQR), 94.4–99.6%}. A negative change in PSA levels from baseline to 12 weeks was observed in only two patients.

**Table 1:** Patient characteristics

Variables		*n* = 60
Age, y, median (IQR)		72.0 (56–93)
ECOG-PS, No. (%)	0	55 (91.7)
	1	5 (8.3)
Baseline PSA level, ng/ml, median (IQR)		195.0 (53.0–610.9)
Baseline Hb level, g/dl, median (IQR)		13.2 (12.0–14.5)
Baseline ALP level, IU, median (IQR)		320.0 (232.0–620.5)
Baseline LDH level, IU, median (IQR)		203.0 (174.0–251.0)
Biopsy Gleason Score, No. (%)	≤8	18 (30.0)
	≥9	42 (70.0)
Site of metstasis, No. (%)	Bone	100 (100)
	Lymphnode	26 (43.3)
	Visceral	2 (3.3)
Presence of bone pain, No. (%)		25 (41.7)
EOD score, No. (%)	1	28 (46.7)
	2	7 (11.7)
	3	11 (18.3)
	4	9 (15.0)
CHAARTED criteria, No. (%)	unknown	5 (8.3)
	low	25 (41.7)
	high	30 (50.0)
	unknown	5 (8.3)
PSA level at 12 weeks, ng/ml, median (IQR)		2.6 (0.5–9.3)
Hb level at 12 weeks, g/dl, median (IQR)		12.5 (11.3–13.3)
ALP level at 12 weeks, IU, median (IQR)		284.0 (233.5–536.0)
LDH levelat 12 weeks, IU, median (IQR)		198.0 (177.0–247.5)
PSA change (baseline-12wks), ng/ml, median (IQR)		159.5 (34.4–546.9)
PSA change at 12 weeks,%, median (IQR)		98.5 (94.4–99.6)

*IQR* Interquartile range, *ECOG* Eastern Cooperative Oncology Group, *PSA* prostate specific antigen, *Hb* hemoglobin, *ALP* alkaline phosphatase, *LDH* lactate dehydrogenase, *EOD* extent of bone disease

**Fig. 1:**
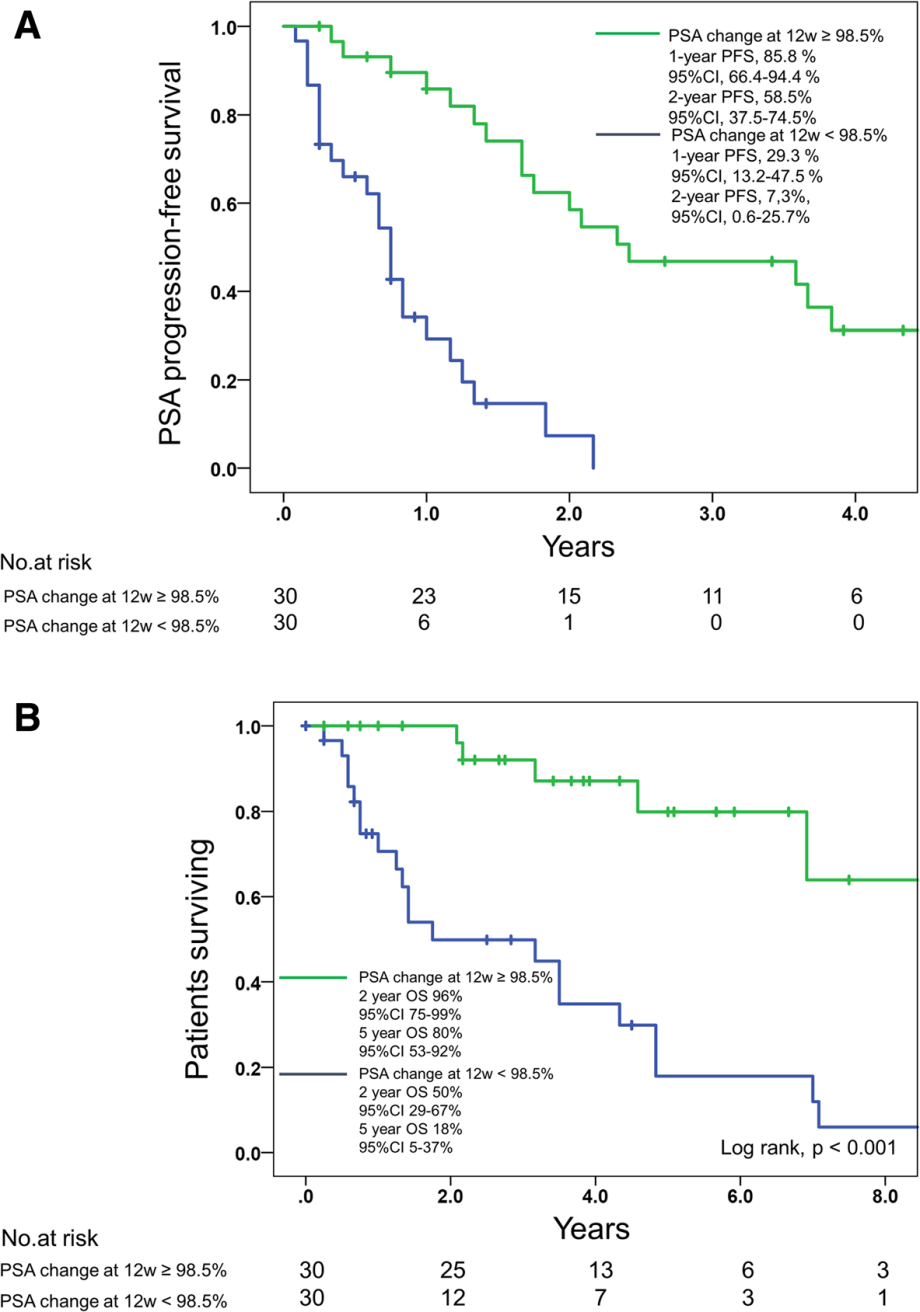
**a** Time to PSA progression-free survival according to the percentage change in the PSA level at 12 weeks. **b** Overall survival according to the percentage change in the PSA level at 12 weeks

**Fig. 2:**
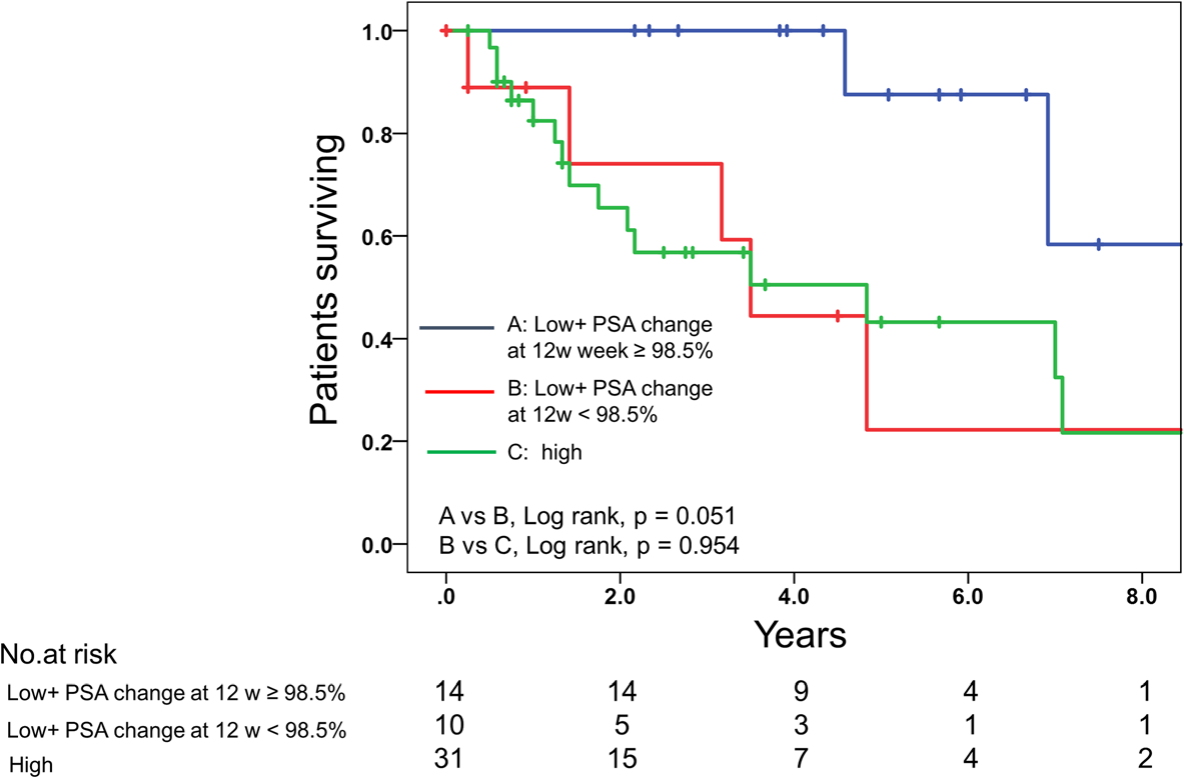
Comparison of overall survival in patients with dichotomized according to subgroups in the CHAARTED trial and percentage change in the PSA level at 12 weeks. The low-volume disease in the CHAARTED trial was dichotomized according to the percentage change in the PSA level at 12 weeks

Using the Kaplan-Meier method to investigate PSA progression-free survival, the data showed that the 1- and 2-year PSA progression-free survival rates of the group of PSA change at 12 weeks of ≥98.5% were markedly higher than those of the group with PSA change at 12 weeks of < 98.5% (85.8% and 58.5 vs 29.3% and 7.3, respectively, *P* < 0.001; Fig. 1a). Using the Kaplan-Meier method to investigate OS (Fig. 1b), the data showed that the 2- and 5-year OS rates of the group with PSA change at 12 weeks of ≥98.5% were markedly higher than the group with PSA change at 12 weeks of < 98.5% (96 and 80% vs 50 and 18%, respectively, *p* < 0.001). Median OS was 103 months for patients with a PSA change at 12 weeks of ≥98.5%, while 38 months for a PSA change at 12 weeks of < 98.5%. When we analyzed the difference in OS based on a percentage reduction in the PSA level for the two subgroup in the CHAARTED trial, OS in patients in the low-volume disease in the CHAARTED trial with a PSA change at 12 weeks of < 98.5% was comparable with that in the high-volume disease (*p* = 0.954; Fig. 2). In addition, patients in the low-volume disease in CHAARTED with a PSA change at 12 weeks of **<** 98.5% tended to have a poor survival compared with those in the low-volume disease in CHAARTED with a PSA change of **≥**98.5% at 12 weeks (*p* = 0.051). The results suggested that the low-volume disease included patients with a poor survival, and that the early change of serum biomarkers might help predict prognosis more precisely.

Results of univariable and multivariable survival analyses using the Cox regression model for PSA progression-free survival are shown in Table 2. The univariable analyses show that the factors significantly associated with PSA progression are a low Hb level at baseline (*p* = 0.001) and at 12 weeks (*p* = 0.043), visceral metastases (*p* = 0.001), an EOD score of ≥3 (*p* < 0.001), a high-volume group in the CHAARTED trial (*p* = 0.007), and a high ALP level at 12 weeks (*p* = 0.001). The best model is shown in Model I selected based on a subset of model selection methods, AIC (i.e., 229.7), and chi-square statistic (i.e., 45.5) which had three variables: a log-transformed baseline PSA level, a log-transformed PSA change, and an EOD score. In the final multivariable model, a log-transformed baseline PSA level (HR 3.62, 95% CI 1.57–8.36) and an EOD score of ≥3 (HR 3.14, 95% CI 1.45–6.82) was statistically associated with increased risk for PSA progression, whereas a one-unit increase in a log-transformed PSA change (baseline-12 weeks) was associated with a decreased risk of PSA progression (HR 0.30, 95%CI 0.13–0.68). To avoid collinearity between a log-transformed baseline PSA level and a log-transformed PSA change, Model II was built where a log-transformed baseline PSA level was removed from Model I that indicated that an EOD score of ≥3 (HR 3.47, 95% CI 1.65–7.33) was an only significant factor associated with increased risk of PSA progression. However, AIC of Model II was higher than Model I and chi-square statistic of Model II was lower than Model I, suggesting that Model I has a better goodness of fit compared to Model II.

**Table 2:** Univariable and multivariable analysis for PSA progression-free survival in patients with mHSPC treated with ADT

					Multivariable					
		Univariable			Model I (Base line PSA adjusted) (*n* = 52; chi-square 45.5; AIC 229.7)			Model II (*n* = 52; chi-square 13.2; AIC 234.7)		
Factors	Group	HR	95% CI	*p* value	HR	95% CI	*p* value	HR	95% CI	*p* value
Age	Continuous	0.99	0.95–1.03	0.642						
ECOG-PS	1 vs 0	1.13	0.40–3.17	0.820						
Log-transformed baseline PSA level (ng/dL)	Continuous	1.14	0.95–1.36	0.156	3.62	1.57–8.36	0.003			
Baseline Hb level (g/dl)	Low vs normal	3.31	1.68–6.51	0.001						
Baseline ALP level (IU/l)	High vs normal	1.58	0.84–2.972	0.157						
Baseline LDH level (IU/l)	High vs normal	1.23	0.66–2.29	0.510						
Presence of bone pain	Yes vs No	1.52	0.81–2.84	0.191						
Biopsy Gleason score	≥8 vs ≤7	2.60	0.62–10.82	0.190						
Visceral metastasis	Yes vs No	28.62	4.03–203.21	0.001						
EOD score	≥3 vs ≤2	3.89	1.91–7.92	<0.001	3.14	1.45–6.82	0.004	3.47	1.65–7.33	0.001
Sub-group in the CHAARTED trial	high vs low	2.44	1.28–4.65	0.007						
Log-transformed PSA change (baseline-12wks)	Continuous	1.10	0.92–1.32	0.281	0.30	0.13–0.68	0.004	1.04	0.87–1.25	0.66
Hb level at 12 weeks	Low vs normal	1.94	1.02–3.67	0.043						
ALP level at 12 weeks	High vs normal	3.19	1.67–6.11	0.001						
LDH level at 12 weeks	High vs normal	1.17	0.59–2.31	0.652						

*mHSPC* metastatic hormone-sensitive prostate cancer, *ADT* androgen deprivation therapy, *ECOG* Eastern Cooperative Oncology Group, *PSA* prostate specific antigen, *Hb* hemoglobin, *ALP* Alkaline phosphatase, *LDH* Lactate dehydrogenase, *EOD* extend of bone metastasis, *HR* hazard ratio, *CI* confidence interbal

Results of the univariable and multivariable survival analyses using Cox regression model for OS are shown in Table 3. Univariable analyses show that the factors significantly associated with a decreased risk for death was a one-unit increase in a log-transformed baseline PSA level (*p* = 0.035) and a log-transformed PSA change (baseline-12 weeks; *p* = 0.014), whereas factors associated with an increased risk for death are bone pain (*p* = 0.049), visceral metastases (*p* = 0.029), an EOD score of ≥3 (*p* = 0.004), a low level group of Hb levels at 12 weeks (*p* = 0.025), and a high level group of ALP levels at 12 weeks (*p* = 0.002). The best model is shown to be Model I, which was selected based on a subset of model selection methods, AIC (i.e.,122.9), and score chi-square value (i.e., 26.5) with five variables of log-transformed PSA- at baseline and PSA change, EOD score, Hb and ALP levels at 12 weeks. In Model I, a high level group of ALP at 12 weeks (HR 3.37, 95% CI 1.01–11.23) was statistically associated with shortened OS time, but no other factors became significant. To avoid the collinearity effect on the OS, Model II was built where a log-transformed baseline PSAlevel was removed from Model I that demonstrated that the *p*-values for a log-transformed PSA change, Hb level at 12 weeks, and ALP level at 12 weeks became closer to significance levels (Hb level was almost significant, but did not reach the significance level). A one-unit increase in a log-transformed PSA change was associated with a decreased risk for OS (HR 0.68, 95% CI 0.54–0.85) whereas a high ALP level at 12 weeks was positively associated with an increased risk for death (HR 3.57, 95%CI 1.11–11.53). The goodness of the model fitness was indistinguishable between Model I (chi-square 26.5; AIC 122.9) and Model II (chi-square 22.8; AIC 121.1).

**Table 3:** Univariable and multivariable analysis for overall survival in patients with mHSPC treated with ADT

					Multivariable					
		Univariable			Model I (Base line PSA adjusted) (*n* = 52; chi-square 26.5; AIC 122.9)			Model II (*n* = 52; chi-square 22.8; AIC 121.1)		
Factors	Group	HR	95% CI	*p* value	HR	95% CI	*p* value	HR	95% CI	*p* value
Age	Continuous	1.01	0.96–1.07	0.702						
ECOG-PS	1 vs 0	1.10	0.26–4.76	0.895						
Log-transformed baseline PSA level (ng/dL)	Continuous	0.81	0.67–0.99	0.035	1.25	0.58–2.71	0.572			
Baseline Hb level(g/dl)	Low vs normal	1.90	0.85–4.27	0.120						
Baseline ALP level (IU/l)	High vs normal	1.34	0.60–3.02	0.480						
Baseline LDH level (IU/l)	High vs normal	1.08	0.48–2.43	0.846						
Presence of bone pain	Yes vs No	2.23	1.00–4.98	0.049						
Biopsy Gleason score	≥8 vs ≤7	0.55	0.16–1.89	0.345						
Visceral metastasis	Yes vs No	5.37	1.18–24.32	0.029						
EOD score	≥3 vs ≤2	3.85	1.55–9.59	0.004	1.97	0.61–6.39	0.256	2.05	0.65–6.46	0.222
Sub-group in the CHAARTED trial	high vs low	2.03	0.86–4.82	0.109						
Log-transformed PSA change (baseline-12wks)	Continuous	0.78	0.64–0.95	0.014	0.55	0.26–1.18	0.123	0.68	0.54–0.85	0.001
Hb level at 12 weeks	Low vs normal	2.44	1.12–5.31	0.025	2.54	0.92–7.00	0.071	2.58	0.95–7.04	0.060
ALP level at 12 weeks	High vs normal	3.47	1.59–7.58	0.002	3.37	1.01–11.23	0.048	3.57	1.11–11.53	0.030
LDH level at 12 weeks	High vs normal	1.74	0.75–4.00	0.197						

*mHSPC* metastatic hormone-sensitive prostate cancer, *ADT* androgen deprivation therapy, *ECOG* Eastern Cooperative Oncology Group, *PSA* prostate specific antigen, *Hb* hemoglobin, *ALP* Alkaline phosphatase, *LDH* Lactate dehydrogenase, *EOD* extend of bone metastasis, *HR* hazard ratio, *CI* confidence interba

## Discussion

In this study, we assessed the prognostic markers for PSA progression-free survival and OS in patients with mHSPC based on early changes in serum biomarkers post-ADT along with the pretreatment risk factors. We found that the level of PSA at baseline as well as PSA change from baseline to 12 weeks were strong prognostic factors of PSA progression. In addition, an EOD score of ≥3 was significantly associated with poor PSA progression free survival. For the risk of death, a large decline in PSA change was associated with a decreased risk, whereas a higher ALP level at 12 weeks was associated with an increased risk. Although the high-volume disease, as defined in the CHAARTED trial, was statistically associated with the PSA progression-free survival, it was not selected in the multivariable model, and was not associated with OS. These findings suggest that the early changes in serum biomarkers have the potential to better predict progression and/or poor prognosis in mHSPC initially treated with ADT compared with the application of only pretreatment variables as predictors.

PSA-related variables, including initial PSA levels and PSA kinetics, have been the most frequently assessed biomarkers in mHSPC [6]. In PSA kinetic variables, PSA nadir and time to PSA nadir are promising biomarkers for mHSPC [12, 13]. However, previous studies reported that the median time to PSA nadir was six to 10 months [12, 13], which means that the prognosis was predictable more than half a year later following the initiation of ADT. In previous phase III trials, ADT had commenced within 120 days before randomization. Therefore, it might be reasonable to predict patients with poor prognosis using early changes (up to three months) in serum biomarkers post-ADT. Park et al. reported that the shorter PSA half-time calculated as log 2 divided by the slope of the linear regression of log PSA vs time using pre- and post-treatment PSA levels assayed every three months were independent risk factors for poor cancer specific survival in patients with mHSPC [8]. A recent study has shown that a velocity of PSA decline > 11 ng/mL per month was significantly associated with an increased risk of progression to castration-resistant prostate cancer after initial ADT [14]. PSA velocity, which is calculated as the slope of log PSA vs. time based on the least squares method using at least two post baseline PSA measurements [15], may achieve a more accurate prediction of outcomes. Although further studies are needed to identify what is the best marker among PSA-related variables to predict the outcome among mHSPC patients, PSA change at 12 weeks is a simple and convenient biomarker for the prediction of clinical outcomes.

Previous studies revealed several cut-off points of the serum PSA level after the administration of ADT to predict the prognosis of mHSPC patients [2, 16]. Hussain et al. investigated prognostic factors based on the absolute PSA level after ADT treatment in 1134 newly-diagnosed cases of mHSPC in the SWOG trial. Their study showed that a PSA of ≤4 ng/mL after 7 months of ADT was a strong predictor of survival [16]. In the phase III trial assessing the impact of concomitant treatment with ADT plus docetaxel in mHSPC reported by Sweeney et al., a complete serological response was defined as a PSA level of ≤0.2 ng/mL [2]. Further studies with a larger number of patients are required because the small number of patients in the dichotomized subgroup is a limitation of our study, although the current study demonstrated that a median percentage of PSA level (98.5%) at 12 weeks was a potential cut-off point of PSA progression and poor survival.

Arai et al. assessed the prognostic factors in 73 non-metastatic and metastatic prostate cancer patients treated with ADT and found that the patients who had a decrease in PSA levels of 80% or more within one month after the beginning of therapy experienced significantly longer free of disease progression [17]. The study was reported at 1990, when the treatment strategy markedly differed from those treated now, and it included the non-metastatic patients. In the current study, 25 patients (41.7%) received docetaxel chemotherapy during their treatment period. Our results suggest that the change in the PSA level at an early stage after the initiation of ADT may have been a risk factor for poor patient outcomes even in recent years when various treatment options including novel anti-androgens, chemotherapies and vaccines became available for advanced prostate cancer.

Regarding other serum biomarkers, Gravis et al. conducted a new prognostic model of mHSPC using a contemporary data set from the phase III study GETUG-15 study [18]. They found that the pre-treatment ALP level was the strongest factor for discriminating patients with a good prognosis; by contrast, in our study, a high level of ALP at 12 weeks was significantly associated with poor OS. Although the Gravis et al. study was a prospective study conducted with a larger number of patients, it could not eliminate the problems such as the impact of upfront docetaxel and/or ADT initiated up to two months before study entry. The combination of pre-treatment variables previously proposed as risk factors of poor OS and the early changes in serum biomarkers evaluated in the current study may be an intriguing option as a novel prognostic model for survival after initial ADT in mHSPC.

Visceral metastasis was one of the well-known prognostic factors for poor OS in metastatic prostate cancer [19]. Regarding the proportion of patients with visceral metastasis, only two (3.3%) patients initially had visceral metastasis in this study, whereas 15.6% of the patients had visceral metastasis at initial diagnosis in the CHAARTED trial [2]. Although the small number of patients with visceral metastasis is a limitation of the current study, OS of the two patients having visceral metastasis were very short (12 month and 21 months). Therefore, the impact of visceral metastases on OS needs to be re-assessed in future study. Severity and location of bone metastasis have also been known to be prognostic factors for poor survivals in a previous study [7]. In our study, the EOD score, which was significantly associated with PSA progression in univariable and multivariable analyses. A recent study showed the impact of more objective scoring of bone metastasis using bone scintigraphy called bone scan index (BSI) [20]. It is intriguing to know the impact of BSI as a novel biomarker in our model because the EOD score tended to be a subjective and semi-quantitative parameter, which may have affected the impact on survivals in the statistical analyses.

Other potential prognostic markers such as serum bone marker [21], circulating tumor cell [22], and single nucleotide polymorphism [23] were also interesting, but not investigated in this study due to lack of adequate data. Further studies should use more sophisticated models using these variables along with the factors established in the current study.

This study has several limitations. First, the major limitations were the retrospective study design. Bias from patients with extremely poor performance status, possibly not be referred to our hospital, which is a tertiary medical center, could have occurred. A larger multicenter prospective study is warranted to validate the results. Second, in spite of the study cohort spanning 15 years, the number of patients we assessed and the number of deaths were small with a short median follow-up. In addition, among our 80 consecutive patients, 20 (25%) patients were excluded from further analysis because of missing laboratory data at 12 weeks after the administration of ADT, thus, further prospective studies are warranted to validate the results using consecutive case series. Lastly, our study did not consider the impact of sequential treatments after initial ADT therapy. Although the continuous and categorical treatment years were not statistically associated with OS in the current study (data not shown), sequential treatments after ADT failure may have a role in patient’s outcome.

## Conclusion

Early changes in serum biomarkers may be associated with early PSA progression-free survival and poor OS in patients with mHSPC. In particular, an increased level of PSA at baseline was an independent predictor of PSA progression, whereas a high level of ALP at 12 weeks was a risk predictor of death for OS. A larger decline in PSA from the baseline until 12 weeks was associated with both PSA progression-free and OS time. The selection of the patients with mHSPC having poor OS by initial ADT using early changes in serum biomarkers, not only pretreatment risk factors, may be useful in adding upfront chemotherapy to these patients.

## Abbreviations

ADT: Androgen deprivation therapy
ALP: Alkaline phosphatese
BSI: Bone scane index
ECOG-PS: Western cooperative oncology group performance status score
EOD score: Extent of bone disease score
Hb: Hemoglobin
MAB: Maximal androgen blockage
mHSPC: metastatic hormone-sensitive prostate cancer
OS: Overall survival
PSA: Prostate specific antigen
